# (5*E*)-5-(4-Meth­oxy­benzyl­idene)-2-(piperidin-1-yl)-1,3-thia­zol-4(5*H*)-one

**DOI:** 10.1107/S1600536811025761

**Published:** 2011-07-06

**Authors:** Hoong-Kun Fun, Chin Sing Yeap, Prajwal L. Lobo, D. Jagadeesh Prasad, Boja Poojary

**Affiliations:** a X-ray Crystallography Unit, School of Physics, Universiti Sains Malaysia, 11800 USM, Penang, Malaysia; b Department of Chemistry, Mangalore University, Karnataka, India

## Abstract

In the title compound, C_16_H_18_N_2_O_2_S, the piperidine ring adopts a chair conformation. The central 4-thia­zolidinone ring makes dihedral angles of 12.01 (7) and 51.42 (9)°, respectively, with the benzene ring and the least-squares plane of the piperidine ring. An intra­molecular C—H⋯S hydrogen bond stabilizes the mol­ecular structure and generates an *S*(6) ring motif. In the crystal, mol­ecules are linked into a tape along the *c* axis by inter­molecular C—H⋯O hydrogen bonds.

## Related literature

For general background to the title compound, see: Lesyk & Zimenkovsky (2004 ▶); Lesyk *et al.* (2007 ▶); Havrylyuk *et al.* (2009 ▶); Ahn *et al.* (2006 ▶); Park *et al.* (2008 ▶); Geronikaki *et al.* (2008 ▶); Zimenkovsky *et al.* (2005 ▶). For hydrogen-bond motifs, see: Bernstein *et al.* (1995 ▶).
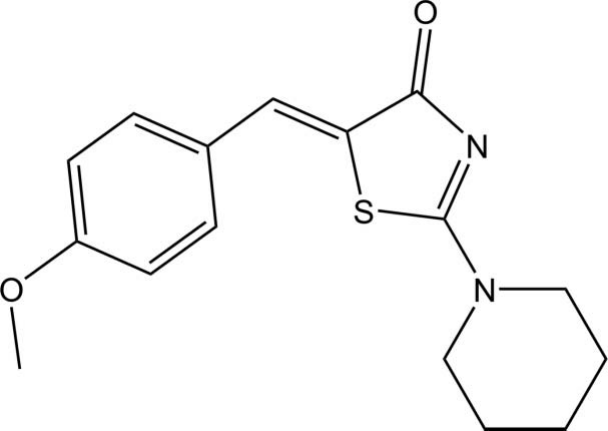


## Experimental

### 

#### Crystal data

C_16_H_18_N_2_O_2_S
*M* *_r_* = 302.38
Monoclinic, 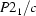

*a* = 8.5811 (3) Å
*b* = 16.5165 (6) Å
*c* = 12.4930 (4) Å
β = 121.518 (2)°
*V* = 1509.42 (9) Å^3^
*Z* = 4
Mo *K*α radiation
μ = 0.22 mm^−1^
*T* = 297 K
0.61 × 0.26 × 0.23 mm

#### Data collection

Bruker APEXII DUO CCD area-detector diffractometer
Absorption correction: multi-scan (*SADABS*; Bruker, 2009 ▶) *T*_min_ = 0.877, *T*_max_ = 0.950
20021 measured reflections
5432 independent reflections
4148 reflections with *I* > 2σ(*I*)
*R*_int_ = 0.023

#### Refinement

*R*[*F* ^2^ > 2σ(*F* ^2^)] = 0.039
*wR*(*F* ^2^) = 0.119
*S* = 1.02
5432 reflections
191 parameters
H-atom parameters constrained
Δρ_max_ = 0.31 e Å^−3^
Δρ_min_ = −0.17 e Å^−3^

### 

Data collection: *APEX2* (Bruker, 2009 ▶); cell refinement: *SAINT* (Bruker, 2009 ▶); data reduction: *SAINT*; program(s) used to solve structure: *SHELXTL* (Sheldrick, 2008 ▶); program(s) used to refine structure: *SHELXTL*; molecular graphics: *SHELXTL*; software used to prepare material for publication: *SHELXTL* and *PLATON* (Spek, 2009 ▶).

## Supplementary Material

Crystal structure: contains datablock(s) global, I. DOI: 10.1107/S1600536811025761/is2743sup1.cif

Structure factors: contains datablock(s) I. DOI: 10.1107/S1600536811025761/is2743Isup2.hkl

Supplementary material file. DOI: 10.1107/S1600536811025761/is2743Isup3.cml

Additional supplementary materials:  crystallographic information; 3D view; checkCIF report

## Figures and Tables

**Table 1:** Hydrogen-bond geometry (Å, °)

*D*—H⋯*A*	*D*—H	H⋯*A*	*D*⋯*A*	*D*—H⋯*A*
C2—H2*A*⋯O1^i^	0.93	2.48	3.3048 (16)	147
C5—H5*A*⋯S1	0.93	2.58	3.2809 (15)	132
C16—H16*A*⋯O1^ii^	0.96	2.48	3.421 (3)	167

Symmetry codes: (i) 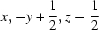; (ii) 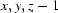.

**Fig. 1.**
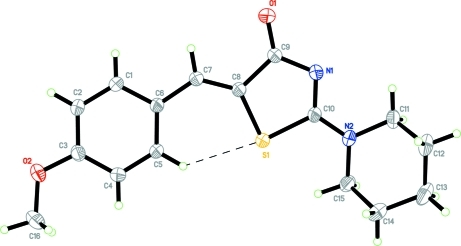
The molecular structure of the title compound, with atom labels and 30% probability ellipsoids for non-H atoms. Hydrogen bonds (dashed lines) are shown.

**Fig. 2.**
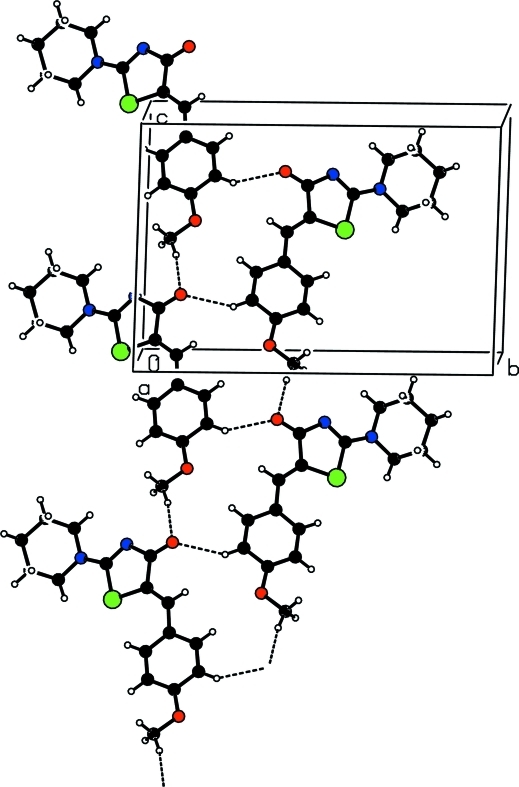
The crystal packing of the title compound, showing the molecules linked along the c axis. Hydrogen bonds (dashed lines) are shown.

## supplementary crystallographic information

### Comment

4-Thiazolidinone ring system is a core structure in various synthetic compounds which display a broad spectrum of biological activities (Lesyk & Zimenkovsky, 2004) including an anticancer effect (Lesyk *et al.*, 2007; Havrylyuk *et al.*, 2009). Mechanisms of 4-thiazolidinones and related heterocycles antitumor activity may be associated with the affinity to anticancer bio-targets, such as phosphatase of a regenerating liver (PRL-3) (Ahn *et al.*, 2006; Park *et al.*, 2008) and non-membrane protein tyrosine phosphatase (SHP-2) (Geronikaki *et al.*, 2008). 5-Arylidene derivatives were previously shown as the most active group of compounds with the anticancer activity among a large pool of 4-azolidone derivatives and analogs (Zimenkovsky *et al.*, 2005). This prompted us to synthesize title compound (I).

The central 4-thiazolidinone ring makes dihedral angles of 12.01 (7) and 51.42 (9)°, respectively, with the benzene ring and the least-squares plane of piperidine ring. The piperidine ring adopts a chair conformation. An intramolecular C5—H5A···S1 hydrogen bond (Table 1) stabilizes the molecular structure and generates an *S*(6) ring motif (Fig. 1; Bernstein *et al.*, 1995). In the crystal structure, the molecules are linked into a tape along the *c* axis by intermolecular C16—H16A···O1 and C2—H2A···O1 hydrogen bonds (Table 1 and Fig. 2).

### Experimental

An equimolar mixture of 2-(piperidin-1-yl)-1,3-thiazol-4(5*H*)-one, anisaldehyde and sodium acetate in acetic acid was refluxed for 2 hrs. The product formed was filtered, washed, dried and re-crystallized from ethanol.

### Refinement

All hydrogen atoms were positioned geometrically (C—H = 0.93–0.97 Å) and refined using a riding model, with *U*_iso_(H) = 1.2 or 1.5*U*_eq_(C). A rotating-group model were applied for methyl group.

### Crystal data

**Table:**

C_16_H_18_N_2_O_2_S	*F*(000) = 640
*M**_r_* = 302.38	*D*_x_ = 1.331 Mg m^−^^3^
Monoclinic, *P*2_1_/*c*	Mo *K*α radiation, λ = 0.71073 Å
Hall symbol: -P 2ybc	Cell parameters from 6389 reflections
*a* = 8.5811 (3) Å	θ = 2.8–32.3°
*b* = 16.5165 (6) Å	µ = 0.22 mm^−^^1^
*c* = 12.4930 (4) Å	*T* = 297 K
β = 121.518 (2)°	Block, brown
*V* = 1509.42 (9) Å^3^	0.61 × 0.26 × 0.23 mm
*Z* = 4	

### Data collection

**Table:**

Bruker APEXII DUO CCD area-detector diffractometer	5432 independent reflections
Radiation source: fine-focus sealed tube	4148 reflections with *I* > 2σ(*I*)
graphite	*R*_int_ = 0.023
φ and ω scans	θ_max_ = 32.6°, θ_min_ = 2.3°
Absorption correction: multi-scan (*SADABS*; Bruker, 2009)	*h* = −12→12
*T*_min_ = 0.877, *T*_max_ = 0.950	*k* = −25→25
20021 measured reflections	*l* = −17→18

### Refinement

**Table:**

Refinement on *F*^2^	Primary atom site location: structure-invariant direct methods
Least-squares matrix: full	Secondary atom site location: difference Fourier map
*R*[*F*^2^ > 2σ(*F*^2^)] = 0.039	Hydrogen site location: inferred from neighbouring sites
*wR*(*F*^2^) = 0.119	H-atom parameters constrained
*S* = 1.02	*w* = 1/[σ^2^(*F*_o_^2^) + (0.0606*P*)^2^ + 0.2092*P*] where *P* = (*F*_o_^2^ + 2*F*_c_^2^)/3
5432 reflections	(Δ/σ)_max_ = 0.001
191 parameters	Δρ_max_ = 0.31 e Å^−^^3^
0 restraints	Δρ_min_ = −0.17 e Å^−^^3^

### Special details

**Table:**

Geometry. All e.s.d.'s (except the e.s.d. in the dihedral angle between two l.s. planes) are estimated using the full covariance matrix. The cell e.s.d.'s are taken into account individually in the estimation of e.s.d.'s in distances, angles and torsion angles; correlations between e.s.d.'s in cell parameters are only used when they are defined by crystal symmetry. An approximate (isotropic) treatment of cell e.s.d.'s is used for estimating e.s.d.'s involving l.s. planes.
Refinement. Refinement of *F*^2^ against ALL reflections. The weighted *R*-factor *wR* and goodness of fit *S* are based on *F*^2^, conventional *R*-factors *R* are based on *F*, with *F* set to zero for negative *F*^2^. The threshold expression of *F*^2^ > σ(*F*^2^) is used only for calculating *R*-factors(gt) *etc*. and is not relevant to the choice of reflections for refinement. *R*-factors based on *F*^2^ are statistically about twice as large as those based on *F*, and *R*- factors based on ALL data will be even larger.

### Fractional atomic coordinates and isotropic or equivalent isotropic displacement parameters (Å^2^)

**Table:**

	*x*	*y*	*z*	*U*_iso_*/*U*_eq_	
S1	0.20003 (4)	0.555618 (16)	0.50684 (2)	0.03982 (9)	
O1	0.39228 (18)	0.38959 (6)	0.75694 (9)	0.0638 (3)	
O2	0.27412 (16)	0.35919 (6)	0.04461 (9)	0.0561 (2)	
N1	0.28424 (15)	0.51913 (6)	0.73753 (9)	0.0425 (2)	
N2	0.16266 (16)	0.64775 (6)	0.66938 (10)	0.0455 (2)	
C1	0.32029 (16)	0.31624 (6)	0.34274 (10)	0.0382 (2)	
H1A	0.3436	0.2711	0.3935	0.046*	
C2	0.30857 (17)	0.30703 (7)	0.22911 (11)	0.0418 (2)	
H2A	0.3216	0.2560	0.2034	0.050*	
C3	0.27723 (16)	0.37401 (7)	0.15287 (11)	0.0409 (2)	
C4	0.2523 (2)	0.44940 (7)	0.19023 (12)	0.0479 (3)	
H4A	0.2292	0.4944	0.1392	0.057*	
C5	0.2618 (2)	0.45754 (6)	0.30353 (12)	0.0457 (3)	
H5A	0.2434	0.5084	0.3271	0.055*	
C6	0.29786 (15)	0.39221 (6)	0.38362 (10)	0.0349 (2)	
C7	0.31827 (16)	0.39806 (6)	0.50618 (10)	0.0374 (2)	
H7A	0.3601	0.3509	0.5536	0.045*	
C8	0.28758 (16)	0.45928 (6)	0.56330 (10)	0.0366 (2)	
C9	0.32720 (18)	0.45128 (7)	0.69525 (11)	0.0422 (2)	
C10	0.21678 (16)	0.57634 (6)	0.65225 (10)	0.0375 (2)	
C11	0.1834 (2)	0.66894 (8)	0.79030 (12)	0.0500 (3)	
H11A	0.2454	0.6255	0.8501	0.060*	
H11B	0.0640	0.6764	0.7798	0.060*	
C12	0.2935 (2)	0.74644 (8)	0.83907 (13)	0.0562 (3)	
H12A	0.2980	0.7631	0.9151	0.067*	
H12B	0.4178	0.7364	0.8601	0.067*	
C13	0.2120 (2)	0.81400 (9)	0.74341 (16)	0.0675 (4)	
H13A	0.2908	0.8611	0.7753	0.081*	
H13B	0.0938	0.8289	0.7301	0.081*	
C14	0.1894 (2)	0.78837 (8)	0.61928 (15)	0.0576 (3)	
H14A	0.1271	0.8307	0.5574	0.069*	
H14B	0.3088	0.7804	0.6300	0.069*	
C15	0.08081 (19)	0.71084 (7)	0.57317 (12)	0.0489 (3)	
H15A	−0.0437	0.7208	0.5517	0.059*	
H15B	0.0770	0.6927	0.4980	0.059*	
C16	0.2685 (3)	0.42727 (11)	−0.02815 (15)	0.0677 (4)	
H16A	0.2886	0.4093	−0.0931	0.102*	
H16B	0.3621	0.4652	0.0252	0.102*	
H16C	0.1511	0.4530	−0.0654	0.102*	

### Atomic displacement parameters (Å^2^)

**Table:**

	*U*^11^	*U*^22^	*U*^33^	*U*^12^	*U*^13^	*U*^23^
S1	0.05518 (18)	0.03126 (13)	0.03620 (14)	0.00014 (10)	0.02609 (12)	−0.00056 (9)
O1	0.1086 (9)	0.0433 (5)	0.0527 (5)	0.0192 (5)	0.0513 (6)	0.0128 (4)
O2	0.0807 (7)	0.0558 (5)	0.0460 (5)	0.0013 (5)	0.0430 (5)	−0.0033 (4)
N1	0.0585 (6)	0.0364 (4)	0.0369 (4)	−0.0010 (4)	0.0280 (4)	−0.0027 (3)
N2	0.0620 (6)	0.0336 (4)	0.0418 (5)	0.0007 (4)	0.0276 (5)	−0.0061 (4)
C1	0.0459 (6)	0.0306 (4)	0.0402 (5)	0.0027 (4)	0.0241 (5)	0.0004 (4)
C2	0.0517 (6)	0.0348 (5)	0.0436 (5)	0.0021 (4)	0.0282 (5)	−0.0047 (4)
C3	0.0470 (6)	0.0423 (5)	0.0392 (5)	−0.0020 (4)	0.0267 (5)	−0.0038 (4)
C4	0.0711 (8)	0.0361 (5)	0.0474 (6)	0.0014 (5)	0.0386 (6)	0.0040 (4)
C5	0.0724 (8)	0.0282 (4)	0.0497 (6)	0.0004 (5)	0.0411 (6)	−0.0009 (4)
C6	0.0410 (5)	0.0307 (4)	0.0364 (5)	−0.0024 (4)	0.0226 (4)	−0.0028 (3)
C7	0.0474 (6)	0.0308 (4)	0.0376 (5)	−0.0014 (4)	0.0248 (4)	−0.0004 (4)
C8	0.0461 (5)	0.0312 (4)	0.0357 (5)	−0.0037 (4)	0.0235 (4)	−0.0020 (4)
C9	0.0594 (7)	0.0352 (5)	0.0380 (5)	0.0000 (4)	0.0297 (5)	0.0006 (4)
C10	0.0447 (5)	0.0320 (4)	0.0364 (5)	−0.0058 (4)	0.0217 (4)	−0.0065 (4)
C11	0.0590 (7)	0.0486 (6)	0.0479 (6)	−0.0020 (5)	0.0319 (6)	−0.0128 (5)
C12	0.0565 (7)	0.0517 (7)	0.0551 (7)	−0.0024 (6)	0.0254 (6)	−0.0216 (6)
C13	0.0740 (10)	0.0401 (6)	0.0770 (10)	0.0010 (6)	0.0316 (8)	−0.0188 (6)
C14	0.0613 (8)	0.0362 (5)	0.0704 (9)	−0.0002 (5)	0.0311 (7)	−0.0004 (6)
C15	0.0561 (7)	0.0361 (5)	0.0490 (6)	0.0042 (5)	0.0236 (6)	−0.0020 (5)
C16	0.0943 (12)	0.0728 (10)	0.0530 (8)	−0.0026 (9)	0.0503 (8)	0.0041 (7)

### Geometric parameters (Å, °)

**Table:**

S1—C8	1.7435 (11)	C7—C8	1.3407 (15)
S1—C10	1.7790 (11)	C7—H7A	0.9300
O1—C9	1.2225 (14)	C8—C9	1.5047 (15)
O2—C3	1.3609 (14)	C11—C12	1.5166 (19)
O2—C16	1.4309 (19)	C11—H11A	0.9700
N1—C10	1.3108 (14)	C11—H11B	0.9700
N1—C9	1.3691 (15)	C12—C13	1.513 (2)
N2—C10	1.3252 (14)	C12—H12A	0.9700
N2—C15	1.4634 (16)	C12—H12B	0.9700
N2—C11	1.4680 (16)	C13—C14	1.519 (2)
C1—C2	1.3781 (16)	C13—H13A	0.9700
C1—C6	1.4053 (14)	C13—H13B	0.9700
C1—H1A	0.9300	C14—C15	1.5096 (18)
C2—C3	1.3912 (16)	C14—H14A	0.9700
C2—H2A	0.9300	C14—H14B	0.9700
C3—C4	1.3852 (16)	C15—H15A	0.9700
C4—C5	1.3811 (17)	C15—H15B	0.9700
C4—H4A	0.9300	C16—H16A	0.9600
C5—C6	1.3919 (15)	C16—H16B	0.9600
C5—H5A	0.9300	C16—H16C	0.9600
C6—C7	1.4506 (15)		
			
C8—S1—C10	88.56 (5)	N2—C11—C12	109.25 (12)
C3—O2—C16	117.82 (11)	N2—C11—H11A	109.8
C10—N1—C9	111.72 (9)	C12—C11—H11A	109.8
C10—N2—C15	124.01 (10)	N2—C11—H11B	109.8
C10—N2—C11	120.94 (10)	C12—C11—H11B	109.8
C15—N2—C11	115.05 (10)	H11A—C11—H11B	108.3
C2—C1—C6	121.52 (10)	C13—C12—C11	111.81 (12)
C2—C1—H1A	119.2	C13—C12—H12A	109.3
C6—C1—H1A	119.2	C11—C12—H12A	109.3
C1—C2—C3	120.09 (10)	C13—C12—H12B	109.3
C1—C2—H2A	120.0	C11—C12—H12B	109.3
C3—C2—H2A	120.0	H12A—C12—H12B	107.9
O2—C3—C4	124.79 (11)	C12—C13—C14	111.12 (11)
O2—C3—C2	115.70 (10)	C12—C13—H13A	109.4
C4—C3—C2	119.52 (10)	C14—C13—H13A	109.4
C5—C4—C3	119.75 (11)	C12—C13—H13B	109.4
C5—C4—H4A	120.1	C14—C13—H13B	109.4
C3—C4—H4A	120.1	H13A—C13—H13B	108.0
C4—C5—C6	122.21 (10)	C15—C14—C13	110.46 (13)
C4—C5—H5A	118.9	C15—C14—H14A	109.6
C6—C5—H5A	118.9	C13—C14—H14A	109.6
C5—C6—C1	116.88 (10)	C15—C14—H14B	109.6
C5—C6—C7	124.46 (9)	C13—C14—H14B	109.6
C1—C6—C7	118.64 (9)	H14A—C14—H14B	108.1
C8—C7—C6	131.54 (10)	N2—C15—C14	110.80 (11)
C8—C7—H7A	114.2	N2—C15—H15A	109.5
C6—C7—H7A	114.2	C14—C15—H15A	109.5
C7—C8—C9	121.47 (10)	N2—C15—H15B	109.5
C7—C8—S1	129.49 (9)	C14—C15—H15B	109.5
C9—C8—S1	109.03 (7)	H15A—C15—H15B	108.1
O1—C9—N1	124.43 (11)	O2—C16—H16A	109.5
O1—C9—C8	122.07 (10)	O2—C16—H16B	109.5
N1—C9—C8	113.49 (9)	H16A—C16—H16B	109.5
N1—C10—N2	123.60 (10)	O2—C16—H16C	109.5
N1—C10—S1	117.18 (8)	H16A—C16—H16C	109.5
N2—C10—S1	119.22 (9)	H16B—C16—H16C	109.5
			
C6—C1—C2—C3	1.20 (18)	C7—C8—C9—O1	1.5 (2)
C16—O2—C3—C4	8.6 (2)	S1—C8—C9—O1	−178.96 (12)
C16—O2—C3—C2	−171.27 (13)	C7—C8—C9—N1	−178.78 (11)
C1—C2—C3—O2	177.84 (11)	S1—C8—C9—N1	0.74 (13)
C1—C2—C3—C4	−2.01 (19)	C9—N1—C10—N2	179.29 (12)
O2—C3—C4—C5	−178.76 (13)	C9—N1—C10—S1	−1.17 (14)
C2—C3—C4—C5	1.1 (2)	C15—N2—C10—N1	−177.59 (12)
C3—C4—C5—C6	0.7 (2)	C11—N2—C10—N1	2.90 (19)
C4—C5—C6—C1	−1.5 (2)	C15—N2—C10—S1	2.88 (17)
C4—C5—C6—C7	176.81 (12)	C11—N2—C10—S1	−176.63 (9)
C2—C1—C6—C5	0.53 (17)	C8—S1—C10—N1	1.37 (10)
C2—C1—C6—C7	−177.87 (11)	C8—S1—C10—N2	−179.07 (10)
C5—C6—C7—C8	9.2 (2)	C10—N2—C11—C12	123.69 (13)
C1—C6—C7—C8	−172.51 (12)	C15—N2—C11—C12	−55.87 (15)
C6—C7—C8—C9	−177.40 (11)	N2—C11—C12—C13	53.89 (16)
C6—C7—C8—S1	3.2 (2)	C11—C12—C13—C14	−54.80 (18)
C10—S1—C8—C7	178.38 (12)	C12—C13—C14—C15	54.15 (17)
C10—S1—C8—C9	−1.08 (9)	C10—N2—C15—C14	−122.75 (14)
C10—N1—C9—O1	179.94 (14)	C11—N2—C15—C14	56.79 (16)
C10—N1—C9—C8	0.25 (15)	C13—C14—C15—N2	−54.03 (16)

### Hydrogen-bond geometry (Å, °)

**Table:**

*D*—H···*A*	*D*—H	H···*A*	*D*···*A*	*D*—H···*A*
C2—H2A···O1^i^	0.93	2.48	3.3048 (16)	147
C5—H5A···S1	0.93	2.58	3.2809 (15)	132
C16—H16A···O1^ii^	0.96	2.48	3.421 (3)	167

Symmetry codes: (i) *x*, −*y*+1/2, *z*−1/2; (ii) *x*, *y*, *z*−1.
